# Influence of grit and healthy lifestyle behaviors on anxiety and depression in US adults at the beginning of the COVID-19 pandemic: Cross-sectional study

**DOI:** 10.34172/hpp.2022.10

**Published:** 2022-05-29

**Authors:** Mike Toczko, Justin Merrigan, Ali Boolani, Bishop Guempel, Italia Milani, Joel Martin

**Affiliations:** ^1^Sports Medicine Assessment Research & Testing (SMART) Laboratory, George Mason University, Virginia, USA; ^2^Human Performance Innovation Center, Rockefeller Neuroscience Institute, West Virginia University, West Virginia, USA; ^3^Department of Physical Therapy, Clarkson University, Potsdam, New York, USA; ^4^Department of Biology, Clarkson University, Potsdam, NY, USA; ^5^Department of Psychology, Clarkson University, Potsdam, NY, USA

**Keywords:** Physiopathology, Health, Physiology

## Abstract

**Background:** The coronavirus disease 2019 (COVID-19) pandemic altered lifestyles and impacted mental health of many adults. Engaging in physical activity, avoiding prolonged sitting, and consuming a healthy diet improve mental health. The current study investigated the association between health-related lifestyle behaviors on feelings of anxiety and depression in adults during the early stages of the COVID-19 pandemic.

**Methods:** Adults (n=796) living in the United States completed an internet-based survey in Spring 2020 that included validated survey instruments for moods, physical activity, sitting and dietary behaviors. Multivariate multiple regression models were used to assess the association between health-related lifestyle behaviors and feelings of anxiety and depression.

**Results:** A majority (70.7%; 95% CI: [0.607, 0.807]) of participants met physical activity (PA) guidelines, 43.7% (95% CI: [0.287, 0.587]) sat for ≥ 8 hours per day, and 87.7% (95% CI [0.807, 0.947]) ate a healthy diet. Our final models explained 6.2% and 9.8% of the variance in anxiety and depression, respectively. Vigorous PA (anxiety: B=-0.111, 95% CI: [-0.171,0.000]; depression: B=-0.111, 95% CI: [-0.186,-0.037]) and dietary behaviors (anxiety: B=-0.112, 95% CI: [-0.180,-0.444]; depression: B=-0.112, 95% CI: [-0.213,-0.076]) were associated with reduced feelings of anxiety and depression while sitting time (anxiety: B=0.119, 95% CI: [0.000,0.199]; depression: B=0.119, 95% CI: [0.199,0.199]) were associated with greater feelings of anxiety and depression.

**Conclusion:** Engaging in vigorous physically activity, reducing sitting time, and consuming a healthy diet was associated with reduced feelings of anxiety and depression during the early part of the pandemic. The aforementioned modifiable lifestyle behaviors are independent of each other suggesting improvements in one behavior may improve feelings of anxiety and depression.

## Introduction

The novel coronavirus (COVID-19) pandemic has disrupted the lives of millions, particularly during surges of COVID-19 cases and hospitalizations when physical distancing was mandated or encouraged to slow the virus’s spread. A consequence of physical distancing intended to mitigate the spread of the COVID-19 virus is decreased socialization with family, friends and co-workers. Thus, as an unintended consequence of physical distancing appears to be worsened mental health,^1^ such as increased symptoms of depression and anxiety.^2^ Previous literature supports that engaging in healthy lifestyle behaviors, such as increasing physical activity (PA),^3^ reducing sitting time,^4^ and eating a healthy diet,^5^ may improve mental health.^6^

It is well established that PA is associated with improved quality of life and reductions in negative mood states.^7^ Furthermore, the intensity of PA appears to be an important factor with moderate-to-vigorous intensity PA (MVPA) potentially more beneficial than light intensity PA to improve mental health.^8^ An unintended consequence of physical distancing appears to be a decline in PA from pre-COVID PA levels, which may negatively influence mental health according to a study including 3052 US adults.^9^ This is particularly concerning as pre-COVID-19 PA data indicated ~80% of adults and adolescents in the United States did not meet PA guidelines of at least 150 to 300 minutes per week of moderate-intensity, or 75 to 150 minutes a week of vigorous-intensity, or a combination of MVPA.^7^ However, the benefits of PA are independently associated with sitting time, meaning each behavior has unique influence with mental health.^10,11^ Sitting for prolonged periods of time is associated with poor mental health,^12^ which is concerning as daily time spent sitting has increased amidst COVID-19.^9,13^ In fact, 42.6% of US adults working from home have reported sitting > 8 hours per day.^13^ Based on prior findings those who are meeting the PA guidelines may not attain the full physiological benefits if engaging in > 8 hours of sedentary behaviors per day.^14^

Eating a healthy diet is another modifiable lifestyle behavior associated with reductions of negative mood states according to a recent meta-analysis of randomized controlled trials of dietary interventions on nonclinical depressive symptoms.^5^ Overall dietary recommendations to improve mood states consists of a diet that includes fruits, vegetables, legumes, nuts, foods rich in omega-3 polyunsaturated fatty acids.^15-17^ While for some individuals COVID-19 has negatively impacted PA related behaviors, several studies have found that dietary behaviors have improved during the pandemic due to individuals eating out less and cooking more meals at home compared to pre-pandemic behaviors.^18,19^ Thus some of the detrimental effects of worsened PA related behaviors may be lessened by improved dietary behaviors during the COVID-19 pandemic.

A meta-analysis by Strickhouser et al suggests that personality is predictive of overall health and well-being.^20^ While several recent studies have investigated the role of healthy lifestyle behaviors influence on mood states during the pandemic they did not account for individual personality traits.^21,22^ The trait of grit has been associated with maintaining PA levels when facing adversity.^23-25^ For example, early in the pandemic, individuals who were ‘grittier’ were more physically active, sat less, and ate healthier diets than those with lower self-perceived grit.^19^ Thus, it seems plausible that during the COVID-19 pandemic grit may be a desirable personality trait associated with lower levels of anxiety and depression. The main purpose of this study was to examine the influence of healthy lifestyle behaviors (PA, sitting time, and diet) on feelings of anxiety and depression while accounting for the personality trait of grit in US adults during the COVID-19 pandemic.

## Material and Methods

### Design

The current study employed a cross-sectional design on data collected from April 13^th^to May 6^th^, 2020 via electronic surveys (Qualtrics Software, XM Provo, UT) as part of a larger investigation on moods and lifestyle behaviors during the COVID-19 pandemic.

### Participants

Researchers aimed to recruit a large sample (n > 1000) of male and female adults aged 18 to 65 years old living in the United States. Due to the rapid changes in living conditions at this time during the COVID-19 pandemic we used a variety of recruitment strategies intended to maximize our sample size. Participants were recruited through mass email, social media (Facebook, Twitter), media publications, and snowball sampling. A link to an anonymous electronic survey was sent to interested participants, after which consent was obtained.

### Instruments

Participants self-reported their age, sex, gender, ethnicity, education, relationship status, household census, residence location, employment (Yes/No, if yes essential/non-essential), smoking, chronic medical conditions, and whether they or anyone they knew had been diagnosed with COVID-19. From the responses dichotomous variables were created for relationship status (committed = married or long-term relationship); education (less educated = no college degree) and employment (employed = currently working full- or part-time). Geo-tracking data were used with data collected from the Johns Hopkins University COVID-19 tracking website to obtain local infection rates for the last 7 days prior to the day that the survey was completed. Infection rates are reported per million.

A modified version of the Profile of Mood States Short Form (POM-SF) was used to measure mood states over the previous 7-day period.^26^ The POM-SF was modified by using a visual analog scale (VAS) to increase the sensitivity to change (0 = “not at all” to 100 = “most extreme”). The current study focused on anxiety (tense + shaky + uneasy + nervous + anxious) and depression (sad + unworthy + discouraged + lonely + gloomy) mood states. Thus, each mood state (anxiety and depression) ranged from 0 to 500. For the current study, POMS-SF had a Cronbach’s alpha of 0.811 and 0.809 for anxiety and depression, respectively. The POM-SF has been found to be a valid and reliable measure of mood states.^27^

Participants were asked to self-report PA through the International Physical Activity Questionnaire-Short Form (IPAQ-SF), a 7-item scale including the frequency (exercise sessions per week), duration (minutes per session), intensity (light, moderate, vigorous), and time spent seated (hours and minutes) over the previous seven-day period.^28^ PA across intensities was categorized as inactive (0 minutes), insufficiently active (0 to < 150 minutes), sufficiently active (150 to < 300 minutes), and highly active ( > 300 minutes). Time spent sitting was categorized as < 4 hours, 4 to < 6 hours, 6 to < 8, and > 8 hours.^29^ The IPAQ-SF has a moderate to high degree of reliability with interclass correlation coefficients between 0.71-0.89 has been reported to be a valid subjective measure of habitual PA.^28,30^

Dietary behaviors were assessed via the Rapid Eating Assessment for Participants Short Version (REAP-S), a 16-item questionnaire including 13 items addressing first part frequency of food choices (i.e., In an average week how often do you: Eat less than 2 servings of fruit a day?) and 3 items addressing the will to change dietary behaviors.^31^ The first 13-items are scored 1 to 3 (1 = usually/often, 2 = sometimes, 3 = rarely/never/does not apply to me). Higher summation of the first 13-items were indicative of healthier diets. Participants reporting < 5 scores of 1 were categorized as having good diets and participants with ≥5 scores of 1 were categorized as having poor diets. The REAP-S questionnaire has good test-retest reliability and is a valid instrument when compared with the Healthy Eating Index (r = 0.472, *P* < 0.001) for measuring dietary behavior.^31,32^

The 8-item short grit scale (Grit-S) was used to measure grit, defined as perseverance and passion towards long-term goals.^33^ Participants responded to eight items on a 5-point Likert scale (1 = Not at all like me, 5 = Very much like me). An aggregated score was divided by 8 to determine total grit scores ranging from 1 (not at all gritty) to 5 (extremely gritty). The Grit-S has been found to be a valid and reliable measure among a diverse populations.^34^ The Cronbach’s alpha for the current study was 0.847.

### Statistical analyses

Data are presented as mean ± standard deviation unless otherwise specified. Due to positively skewed distributions, independent lifestyle variables were winsorized to mitigate the influence of extreme values.^35^ Winsorizing involves taking extreme values and replacing them with the value that corresponds with a respective percentile of the original distribution. Due to the skewness of our data extreme values were winsorized to the 95^th^ percentile. The dependent mood variables of anxiety and depression were also positively skewed. In order to address the skewness both dependent variables were transformed by taking the square root and then each outcome was standardized to ensure variables were on the same scale. Univariate outliers were removed if participants were > 3 standard deviations above or below the mean for each variable. The assumption of multi-collinearity was met based on bivariate correlations^36^ between predictors and the variance inflation factor ( < 2.5).

Multivariate multiple linear step-wise regression models were used to examine the association of modifiable lifestyle behaviors (PA, sitting time, diet) on anxiety and depression (dependent variables). In the first step grit and demographic variables (sex, smoking, chronic medical conditions, employment status, living in a populated area, relationship status and 7-day infection rates) were included as independent variables to control for confounding factors reported to influence moods during the COVID-19 pandemic.^37,38^ The models controlled for infection rates since a recent study reported that COVID-19 infection rates were associated with mental health during the current COVID-19 pandemic.^39^ A second regression model was computed with lifestyle behaviors (PA, sitting time, and diet) added to examine the added effects of these variables on anxiety and depression. The observed power for each model reported in the results was computed using a T-family test based on sample size, number of predictors and observed R^2^. All analyses were completed using SPSS v26.0 (IBM Corp. Released 2016, IBM SPSS Statistics for Windows: Armonk, NY), significance level was set at α = 0.05.

## Results

The sample (n = 796) consisted of 583 females (73.2%) and 213 males (26.8%) who were well educated, 37% completed a bachelor’s degree and 40.4% completed a graduate degree. Majority of the sample were free of chronic conditions (72.2%), fully employed (pre-COVID-19: 59.4%; current-COVID-19: 52.1%) and married (59.8%) without children at home (72.2%). The self-reported COVID-19 7-day infection rate was 75.8 ± 178.12. Lifestyle variables are provided in Table 1. Notably, 70.7% of the sample met PA guidelines and 43.7% reported sitting for > 8 hours per day. In the first step of our step-wise linear regression modeling neither multivariate regression models were statistically significant (Anxiety: R^2^ = 0.009, adjusted R^2^ = -0.002, F(9, 786) = 0.832, *P* = 0.586; Depression: R^2^ = 0.014, adjusted R^2^ = 0.003, F(9, 786) = 1.234, *P* = 0.270). In the second step (including addition of modifiable lifestyle factors) of multivariate regression modeling our models for both anxiety and depression were statistically significant (Anxiety: R^2^ = .061, adjusted R^2^ = 0.044, F(14, 781) = 3.478, *P* < 0.001; Depression: R^2^ = 0.094, adjusted R^2^ = 0.077, F(14, 781) = 5.756, *P* < 0.001). Significant predictors of anxiety and depression were education status (Λ = 0.992; F(2, 780) = 3.053; *P* = 0.048; η^2^ = 0.008; = 0.590), vigorous PA (Λ = 0.988; F(2,780) = 4.838; *P* = 0.008; η^2^ = 0.012; = 0.800), sitting time (Λ = 0.972; F(2, 780) = 11.402; *P* < 0.001; η^2^= 0.028; = 0.993), and REAP-S (Λ = 0.979; F(2, 780) = 8.448; *P* < 0.001; η^2^ = 0.021; = 0.965).

**Table 1:** Participant mental health, grit and healthy lifestyle continuous variables

**Variable**	**Mean**± **SD**	**5** ^th^ ** Percentile**	**50** ^th^ ** Percentile**	**95** ^th^ ** Percentile**
Anxiety	176.00 ± 121.66	14.00	150.00	396.00
Depression	157.00 ± 124.56	5.00	129	396.00
Grit	3.45 ± 0.62	2.38	3.50	4.38
Vigorous Physical Activity (min/day)	29.82. ± 37.12	0.00	17.14	137.14
Moderate Physical Activity (min/day)	25.19. ± 34.15	0.00	11.43	120.00
Light Physical Activity (min/day)	49.80. ± 65.03	0.00	25.71	257.14
Meeting Physically Activity Guidelines				
Yes	70.7%	-	-	-
No	29.3%	-	-	-
Physical activity (4 levels)				
Inactive	19.4%	-	-	-
Insufficiently active	12.0%	-	-	-
Sufficiently active	12.6%	-	-	-
Highly active	53.6%	-	-	-
Sitting Time (min/day)	425.13 ± 199.21	102.86	420.00	840.00
Hours of sitting per day (2 levels)				
Low ( < 6 hours)	36.5%			
High ( > 6 hours)	63.5%			
Hours of sitting per day (4 levels)				
0-4 hours	15.8%	-	-	-
4-6 hours	21.7%	-	-	-
6-8 hours	18.8%	-	-	-
8+ hours	43.7%	-	-	-
REAP-S total score	30.30 ± 4.06	23.00	31.00	36.00
REAP-S flags	2.16 ± 1.99	-	-	-
Diet category				
Good	87.7%	-	-	-
Bad	12.3%	-	-	-

Notes: 1. Anxiety and depression ranged from 0 to 500. 2. Meeting physical activity guidelines was determined by whether participants reported greater than 150 minutes of moderate and vigorous physical activity (Active) per week. Participants were further categorized as inactive (0 minutes), insufficiently active (0 to < 150 minutes), sufficiently active (150 to < 300 minutes) and highly active ( > 300 minutes). 3. Diet Categories: Good = REAP-S Flags < 5; Bad = REAP-S Flags > 5 (REAP-S = Rapid Eating Assessment for Participants Short Form). 4. Extreme values ( > 95^th^ percentile) of vigorous physical activity, moderate physical activity, light physical activity, sitting and REAP-S were set equal to the 95^th^ percentile in our multivariate analysis.

The first regression model was not statistically significant for anxiety, but the addition of modifiable lifestyle factors led to a statistically significant overall association with anxiety (∆R^2^ = 0.053, R^2^ = 0.061, *P* < 0.001; Table 2). Similarly, the first regression model was not statistically significant for depression, but the addition of modifiable lifestyle factors led to a statistically significant overall association with depression (∆R^2^ = 0.084, R^2^ = 0.094, *P* < 0.001; Table 2). Greater levels of vigorous PA and better dietary behaviors reduced self-perceived feelings of anxiety and depression, while greater amounts of sitting time increased feelings of anxiety and depression (Table 2). A post-hoc T-family test revealed an observed power of 1.0 for the final models (Table 2); however, for the initial anxiety and depression models the observed power was 0.421 and 0.614, respectively.

**Table 2:** Multiple linear regression models of anxiety and depression

**Predictors**	**Anxiety**				**Depression**			
	**Model 1**		**Model 2**		**Model 1**		**Model 2**	
	**B**	**95% CI**	**B**	**95% CI**	**B**	**CI95%**	**B**	**95% CI**
Age (years)	0.012	(-0.073, 0.085)	-0.012	(-0.085, 0.073)	0.000	(-0.073, 0.085)	-0.012	(-0.085, 0.061)
7-day Average Infection Rate	0.000	(0.000, 0.178)	0.000	(0.000, 0.178)	0.000	(0.000, 0.178)	0.000	(0.000, 0.178)
Sex (ref: male)	0.006	(-0.064, 0.076)	-0.005	(-0.074, 0.063)	0.000	(-0.070, 0.070)	-0.014	(-0.081, 0.054)
Education (ref: less educated)	-0.087^a^	(-0.162, -0.013)	-0.082^a^	(-0.156, -0.009)	-0.095	(-0.169, -0.020)	-0.088^a^	(-0.160, -0.016)
Relationship status (ref: committed)	-0.034	(-0.144, 0.076)	-0.021	(-0.091, 0.048)	-0.103^b^	(-0.213, 0.007)	-0.066^b^	(-0.134, 0.002)
Children living home? (ref: yes)	0.003	(-0.049, 0.056)	0.008	(-0.067, 0.084)	0.021	(-0.031 0.074)	0.035	(-0.039, 0.109)
Chronic medical conditions? (ref: yes)	-0.013	(-0.085, 0.060)	-0.012	(-0.112, 0.080)	-0.033	(-0.105, 0.040)	-0.028	(-0.098, 0.042)
Employment (Ref: employed)	-0.027	(-0.099, 0.044)	-0.028	(-0098, 0.045)	-0.004	(-0.075, 0.067)	-0.002	(-0.075, 0.071)
Grit	-0.022	(-0.095, 0.051)	-0.019	(-5.38, 3.16)	0.020	(-0.053, 0.092)	0.023	(-0.047, 0.093)
VPA (min/day)	-	-	-0.111^a^	(-0.171, 0.000)	-	-	-0.111^c^	(-0.186, -0.037)
MPA (min/day)	-	-	0.000	(0.195, 0.130)	-	-	0.034	(-0.034, 0.102)
LPA (min/day)	-	-	0.000^a^	(-1.99, 0.398)	-	-	0.000	(-0.065, 0.065)
Sitting Time (min/day)	-	-	0.199^d^	(0.000, 0.199)	-	-	0.199^d^	(0.199, 0.199)
REAP-S score	-	-	-0.112^c^	(-0.180, -0.044)	-	-	-0.144^d^	(-0.213, -0.076)
**Model**								
F	0.832		3.38		1.234		5.756	
p-value	0.586		< 0.001		0.270		< 0.001	
R^2^	0.009		0.061		0.014		0.094	
Adjusted R^2^	-0.002		0.044		0.003		0.077	
∆R^2^	-		0.052		-		0.080	
Observed Power	0.421		1.0		0.614		1.0	

Notes: 1. Model 1 included grit and demographic predictors only; Model 2 included the same predictors as model 1 with lifestyle predictors added (i.e. physical activity and diet) 2. Abbreviations: 95% CI, 95% confidence interval; REAP-S, Rapid Eating Assessment for Participants Short Form; VPA, vigorous physical activity; MPA, moderate physical activity; LPA, light physical activity. 3. Predictor values are standardized beta; ^a^* P* < 0.05; ^b^*P* < 0.1; ^c^* P* < 0.01; ^d^* P* < 0.001. Post-hoc power was computed using a T-family test.

## Discussion

This study aimed to understand the association between PA, total time spent sitting, and dietary behaviors on anxiety and depression while accounting for the personality trait of grit in US adults during the COVID-19 pandemic. Our findings suggest that engaging in more PA, less total time spent sitting, and improving diet are associated with lower levels of anxiety and depression during early stages of the COVID-19 pandemic. Previous literature during early stages of COVID-19 pandemic has shown reduced PA and increased time spent sitting, compared to levels prior to the pandemic, which may exacerbate negative mental health effects of physical distancing.^9,13^ Yet, greater levels of PA has previously shown to be associated with improved feelings of anxiety and depression.^3,40^ Further, PA intensity appeared important, as vigorous PA was a significant predictor of anxiety and depression and light and moderate PA were not. These findings are in agreement with previous literature prior to the COVID-19 pandemic that found moderate to vigorous levels of PA were more associated with fewer symptoms of anxiety and depression.^8,41^

In the current study total time spent sitting was inversely associated with levels of anxiety and depression. Prior literature suggests that this association may be bi-directional, meaning that sedentary behavior can be a symptom or a cause of poor mental health.^42-44^ Prior to the COVID-19 pandemic, the US was facing a sedentarism pandemic,^45^ which has generally been worsened due to the COVID-19 pandemic from shelter in place and physical distancing protocols.^9,44^ The continued reductions in social interaction may influence PA compounding the existing low PA levels and high sedentary behavior.^45^ Unfortunately, PA and sedentary behavior are independently associated with mental health, improving PA adherence may not nullify the negative effects sustained from high sedentary behavior.^4^

Approximately 85%-90% of our sample reported good dietary behaviors and the regression results indicated that anxiety and depression decreased with improved dietary behaviors. The high degree of good dietary behaviors suggests that many individuals were eating healthy at the onset of the pandemic and could be due to reductions in available restaurants and fast food, more time was spent cooking at home.^18^ Recent literature has shown that lower diet quality increased symptoms of anxiety and depression,^46^ while dietary interventions have shown to reduced negative mood states.^47^ Suggesting that regardless of PA levels, dietary behaviors may have a large influence on mood states specifically during adverse times.

Our findings suggested that grit was not associated with anxiety or depression during the time our data was collected during the onset of the COVID-19 pandemic. These findings were unexpected based on previous literature which has found a beneficial relationship between high levels of grit and depression but limited to no relationship between grit and anxiety.^48^ A suggested “zone” of anxiety may be beneficial during the COVID-19 pandemic characterized by negative consequences for one’s health if too little or too much anxiety is present. This suggests that the linear regression models within our study may not have been an appropriate choice for detecting an association. Additionally, grit and depression are characterized by opposing feelings.^49^ Depression being characterized as amotivation and a sense of hopelessness,^49,50^ while grit is measured by the motivation to overcome adversity.^33,50^ This distinction would suggest that those with higher levels of grit may experience less feelings of depression during pandemic conditions due to their inherent motivation. Ultimately, we found grit to not significantly influence anxiety or depression. We believe this finding indicates that enhancement of healthy lifestyle behaviors such as vigorous PA, and diet were of greater importance in the reduction of negative mood states. While greater durations of sitting time is suggested to exacerbate feelings of anxiety and depression.

The strengths of the current study include controlling for COVID-19 infection rates in the multivariate regression model and the statistical approach. Infection rates have been reported to be associated with mental health during the current COVID-19 pandemic^39^ yet many of the prior studies examining the role of healthy lifestyle behaviors on mental health neglect to account for this confounding factor.^21,22^ Our multivariate analysis allowed us to minimize type I error while being able to simultaneously compare healthy lifestyle behaviors for feelings of anxiety and depression. Limitations were encountered within our study. Firstly, the use of a cross-sectional design allows for suggestive treatments but does not allow for a true causal relationship.^51^ Secondly, males and females were not equally distributed across all age groups. Most groups had a distribution of two-thirds female to male ratios. Self-reported responses may have been inflated or reported incorrectly.^52^ Prior literature specifically shows that when individuals are tasked with self-reporting PA, the inputs can be over or underestimated.^53^ The cut point bias hypothesis has been proposed,^52^ which suggests that self-reported methods will overestimate moderate to vigorous PA due to variations in energy expenditure between and within individuals as well as total time spent within activities at the three MET’s threshold.^52^ Lastly, our regression models were relatively weak in several regards. Our initial models had low observed power which can be attributed to p-values substantial above the 0.05 significance level.^54^ Additionally, the variance explained within our models was small, indicating additional variables should be explored to strengthen what was observed.

## Conclusion

The COVID-19 pandemic continues to facilitate sedentary behavior through self-isolation, social distancing, and working from home. Negative mental health effects have become more prevalent due to this increase in sedentary behavior.^9^ Our findings establish an emphasis to participate in daily vigorous PA, consume a healthy diet, and reduce total time spent sitting to improve anxiety and depression levels during the COVID-19 pandemic. Notably PA, total time spent sitting and diet are three distinct and independently modifiable behaviors.^4,47^ Thus we suggest a holistic approach be taken characterized by emphasizing improvements to all behaviors provide the best results in decreasing anxiety and depression levels. Improvement to only one behavior may not elicit beneficial changes in negative mood states. Future research should explore changes in our reported relationships over time throughout the pandemic as these relationships likely changed as the pandemic progressed.

## Authors’ contributions

MT:Data analysis, writing, revising and approved the final manuscript. JM: Data analysis, writing, revising and approved the final manuscript. AB: Conceptualization and design, writing, revising and approved the final manuscript. BG: Data analysis, writing and approved the final manuscript. IM: Data preparation and approved the final manuscript. JM: Conceptualization and design, data analysis, writing, revising and approved the final manuscript.

## Funding

No funding was received.

## Ethical approval

The study involving human participants were reviewed and approved by George Mason University (#20.5-1) and Clarkson University (#15922393-1). The participants provided their informed consent prior to participation in this study.

## Competing interests

None declared.
